# A Review of the Potential of Phytochemicals from* Prunus africana* (Hook f.) Kalkman Stem Bark for Chemoprevention and Chemotherapy of Prostate Cancer

**DOI:** 10.1155/2017/3014019

**Published:** 2017-02-13

**Authors:** Richard Komakech, Youngmin Kang, Jun-Hwan Lee, Francis Omujal

**Affiliations:** ^1^University of Science & Technology (UST), Korean Medicine Life Science, Daejeon 34054, Republic of Korea; ^2^Korea Institute of Oriental Medicine (KIOM), Yuseongdae-ro, Yuseong-gu, Daejeon 34054, Republic of Korea; ^3^Natural Chemotherapeutics Research Institute (NCRI), Ministry of Health, P.O. Box 4864, Kampala, Uganda; ^4^Makerere University, P.O. Box 7062, Kampala, Uganda

## Abstract

Prostate cancer remains one of the major causes of death worldwide. In view of the limited treatment options for patients with prostate cancer, preventive and treatment approaches based on natural compounds can play an integral role in tackling this disease. Recent evidence supports the beneficial effects of plant-derived phytochemicals as chemopreventive and chemotherapeutic agents for various cancers, including prostate cancer.* Prunus africana* has been used for generations in African traditional medicine to treat prostate cancer. This review examined the potential roles of the phytochemicals from* P. africana*, an endangered, sub-Saharan Africa plant in the chemoprevention and chemotherapy of prostate cancer. In vitro and in vivo studies have provided strong pharmacological evidence for antiprostate cancer activities of* P. africana*-derived phytochemicals. Through synergistic interactions between different effective phytochemicals,* P. africana* extracts have been shown to exhibit very strong antiandrogenic and antiangiogenic activities and have the ability to kill tumor cells via apoptotic pathways, prevent the proliferation of prostate cancer cells, and alter the signaling pathways required for the maintenance of prostate cancer cells. However, further preclinical and clinical studies ought to be done to advance and eventually use these promising phytochemicals for the prevention and chemotherapy of human prostate cancer.

## 1. Introduction

Prostate cancer is one of the most common nonskin cancers in men. It is caused by unregulated prostate cell division, which leads to abnormal growth, with the potential to spread to other parts of the body [1]. These neoplastic cells originate from highly specialized cells through a process of regression to an advanced stage. Unlike the normal parent cells, these cells divide continuously, resulting in a tumor. Approximately, 9–11% of men are at risk of clinically suffering from prostate cancer in their life time [2–5]. Prostate cancer is typically androgen-dependent during its initial stages when the hormone androgen binds to the androgen receptor (AR) and then transactivates target genes [6, 7]. Androgen and AR-mediated signaling are therefore crucial for the development and functioning of both the normal prostate and prostate cancer. The importance of androgen in prostate cancer is further supported by the fact that prostate cancer rarely occurs in men with the deficiency in 5*α*-reductase, an enzyme that converts testosterone to its active metabolite 5*α*-dihydrotestosterone (DHT) [8]. Currently, one of the main approaches to the treatment of prostate cancer is downregulation of androgens by antiandrogenic agents [5, 6, 9, 10]. For years, prostate cancer, similar to other forms of cancer, has been managed through the conventional treatment modalities such as surgery, radiation therapy, cryosurgery, and hormone therapies [11]. However, there is still no effective treatment for advanced stages of prostate cancer. Prostate cancer has been known to progress slowly and it is crucial to prevent its occurrence to reduce the risk of development of the disease. Chemoprevention and chemotherapy, including the administration of one or more naturally occurring antiprostate cancer agents [1, 3, 4, 10] have been identified as approaches by which the prevalence of such diseases as prostate cancer can be reduced, suppressed, or reversed. In the last decades, several plants have been confirmed to contain chemopreventive and therapeutic agents for various cancers including prostate cancer [10, 12–14]. More importantly, over 60% of currently used anticancer agents are estimated to be from natural sources [13]. Among plants with enormous antiprostate cancer potential is* Prunus africana* (African cherry), which belongs to the plant family Rosaceae. This evergreen miraculous plant is only found in sub-Saharan Africa and is highly sought after owing to its unique anticancer phytochemicals [1, 2, 15]. In fact, the use of* P. africana* in African traditional medicine (ATM) to treat prostate cancer and related conditions is not a new phenomenon across various communities in Africa [1]. More importantly, the use of* P. africana* has been patented in France for prostate cancer treatment [16]. In addition to prostate cancer, the bark extract of* P. africana* has for many years been used for the treatment of benign prostatic hyperplasia (BPH). Recent studies by Nyamai et al. [17] and Jena et al. [18] confirmed the effectiveness of the bark extract of* P. africana* in BPH treatment and attributed this to the synergistic effects of pentacyclic triterpenoids, ferulic esters of long-chain fatty alcohols, and phytosterols contained in* P. africana* bark. The phytosterols (including *β*-Sitosterol) and pentacyclic triterpenoids (including ursolic acids) also have anti-inflammatory effects on the prostate [17]. In ATM,* P. africana* is also used to treat myriad of diseases including but not limited to diarrhea, epilepsy, arthritis, hemorrhage, and hypertension [15, 16, 19–21]. The novel phytochemicals from* P. africana*, suggested for the treatment of prostate cancer are ursolic acid, oleanolic acid, *β*-amyrin, atraric acid (AA), N-butylbenzene-sulfonamide (NBBS), *β*-sitosterol, *β*-sitosterol-3-O-glucoside, ferulic acid, and lauric acid [20, 21]. The use of* P. africana* in cancer chemotherapy and chemoprevention has been discussed in a number of peer reviewed journal articles. This review therefore sought to examine the phytochemicals from* P. africana* that have the potential for prostate cancer chemoprevention and chemotherapy-both in vitro and in vivo with the goal of finding new drugs for prostate cancer.

## 2. Methods

In this review, we modified the data search process used by Kim et al. [47] and Lin et al. [48] to obtain information from original peer reviewed articles published in scientific journals, with a focus on the botany, distribution, and potential of* P. africana* for cancer chemoprevention and chemotherapy. We carefully searched electronic literature databases including but not limited to PubMed, Scopus, and Google Scholar for relevant records for a period from 1995 to 2016. The following key search terms were used (“*P. africana*” OR “African cherry” OR “*Pygeum africanum*” AND “Prostate cancer” OR “Distribution” OR “Phytochemicals”) OR (“Phytochemicals in* P. africana*” AND “Prostate cancer”), OR (“Chemotherapy” OR “Chemoprevention” OR “Treatment” AND “Prostate cancer”) OR (“African traditional medicine” AND “Prostate cancer”) OR (“*Prunus africana* phytochemicals” AND “Apoptosis” OR “Androgen receptors” OR “Cell proliferation” OR “Anti-prostate cancer properties”). The data obtained were verified independently for their accuracy and any inconsistencies were settled through discussions between the authors. The final data obtained through discussions among the authors were then summarized, analyzed, and compared, and conclusions were made accordingly.

## 3. *Prunus africana* Botany and Distribution

**Figure 1:**
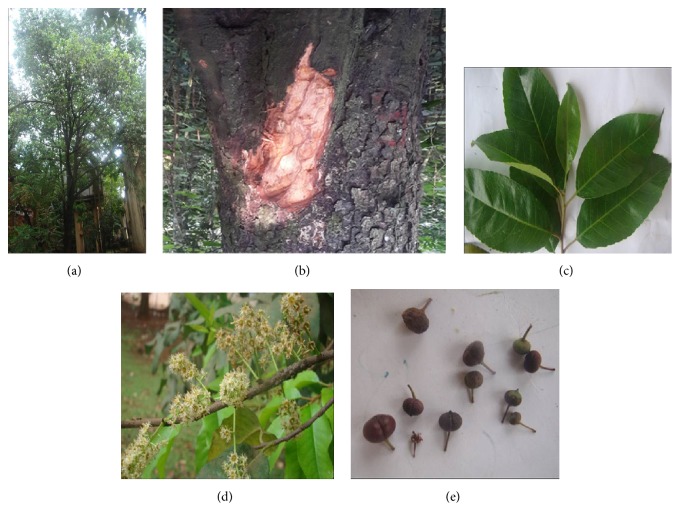
The botanical morphology of the main features of* P. africana*. (a)* P. africana* tree growing at backyard of Natural Chemotherapeutics Research Institute, Uganda. (b) Blackish-brown bark of* P. Africana*. (c) Simple, alternate, oval-shaped, leaf of* P. Africa*. (d) Greenish or white flowers of* P. Africana*. (e) Spherical, pinkish-brown, bilobed, fruit of* P. africana*.

The genus* Prunus* comprises over 400 species, of which only 98 are of great importance [50]. The African cherry is a species of the genus* Prunus,* with a mature stem diameter of up to 1 m and a height of more than 40 m with open branches (Figure 1(a)); and a blackish-brown bark (Figure 1(b)). Leaves are simple, alternate, oval-shaped, shiny-deep green on the top side and lighter on the underside, with a conspicuous prominent midrib on the underside (Figure 1(c)). Flowers are greenish or white (Figure 1(d)), and fruits are spherical, 7 mm long, 1.3 cm wide, pinkish-brown, and bilobed, with thin, dark red to reddish brown pulp when ripe (Figure 1(e)) [20].

**Figure 2:**
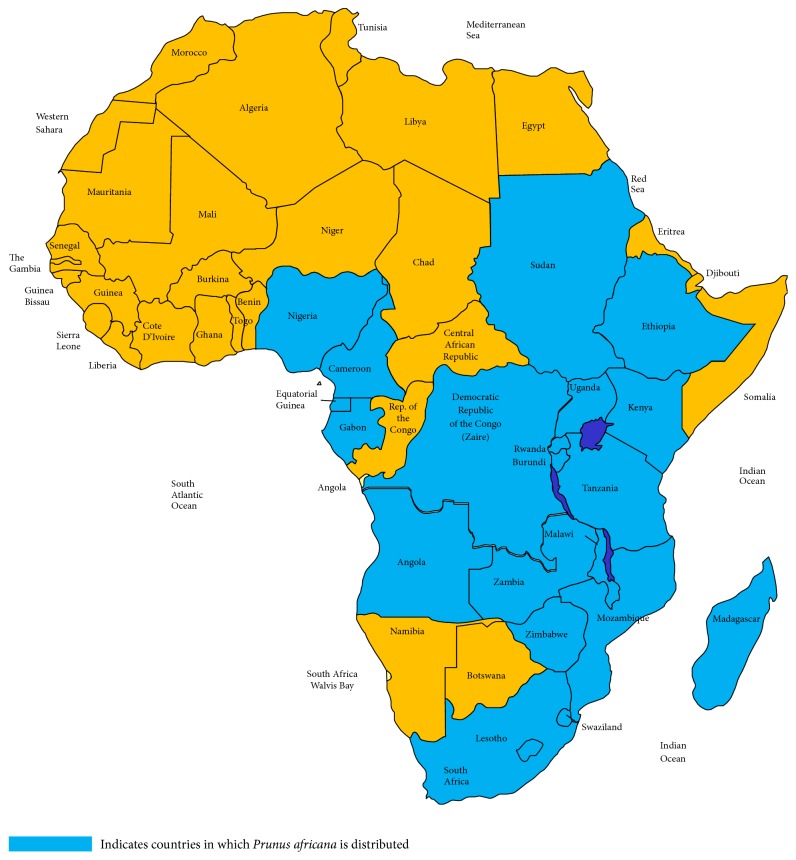
Modified map from Hall et al. [49] on distribution of* P. africana.*

The genus name “*Prunus*” is derived from a Latin word which refers to the plum family, and the scientific name “*Prunus africana*” refers to the species of African origin. This monoecious tree is native to 21 countries in sub-Saharan Africa (Figure 2) [19, 51, 52]. It is a highland forest plant that grows in humid and semihumid conditions at an altitude of about 900–3,400 m above the sea level, with a mean annual rainfall of 890–2,600 mm and a mean annual temperature of 18–26°C [20, 53].

The discovery of the medicinal properties of the* P. africana* bark for a myriad of health conditions initiated a massive harvest of its stem bark for international market needs [54]. To date, there has been an increasing demand for the bark of* P. africana* both locally and internationally, for the production of herbal medicines for the treatment of prostate cancer and related conditions [20]. Unfortunately, in most scenarios, it is the stem bark of the plant that is targeted (Figure 1(b)), which puts the survival of the plant at a great risk, if not done in accordance with the guidelines. In fact, poor harvest practices coupled with overexploitation has severely affected the wild population of* P. africana* [55, 56]. Consequently,* P. africana* has been added to Appendix II of the Convention on International Trade in Endangered Species of Wild Fauna and Flora (CITES) list of endangered species, for the regulation of its trade from wild harvest and all exports of* P. africana* are currently subjected to a CITES export permit to protect the plant from extinction [51].

## 4. In Vitro and In Vivo Effects of Ethanolic Stem Bark Extracts of* P. africana* on Prostate Cancer Cells

*P. africana* is one of the many medicinal plants containing large quantities of bioactive compounds that can be used for prostate cancer management [15, 20, 22, 57]. Previous studies have shown that* P. africana* extracts as an antiprostate cancer treatment targets fast dividing cells by impairing mitosis or by causing target cells to undergo apoptosis [1, 20, 57]. Apoptosis is a biological process that occurs through a series of programmed cell death steps characterized by morphological alterations, including plasma and nuclear membrane blebbing, cell shrinkage, dissolution of the nuclear lamina, and biochemical processes responsible for the activation of apoptosis [58]. In a tissue culture study performed by Shenouda et al. [40], ethanolic extracts of* P. africana* showed growth inhibition of a human prostate cancer cell line (PC-3) and epithelial cells derived from a lymph-node carcinoma of the prostate (LNCaP) by 50% at 2.5 *μ*L/mL and also induced significant apoptosis in both cell lines (PC-3 and LNCaP) at 2.5 *μ*L/mL compared to untreated cells. In an in vivo study using TRAMP (transgenic adenocarcinoma of the mice prostate), a model for the pathogenesis of human prostate cancer, the mice that were fed on* P*.* africana* extract showed a significant reduction (*p* = 0.034) in the prostate cancer incidence (35%) compared to casein fed mice (62.5%) [40]. In another study by Margalef et al. [59], they observed that* P. africana* ethanolic extract had an antimitogenic effect on prostate cancer cells by inhibiting the mitogenic action of epidermal growth factor which resulted in a decreased number of cells entering the S-phase of the cell cycle. Thus, preclinical findings have shown that* P. africana* has a large potential in the regulation of prostate cancer by inhibiting the growth of prostate cancer cell lines and causing apoptosis both in vitro and in vivo. Hence,* P. africana* phytochemicals can be used as an effective cytotoxic chemotherapy in the treatment of men with prostate cancer and other prostate related conditions.

## 5. Pharmacological Efficacy of Antiprostate Cancer Phytochemicals from* P. africana*

The antiprostate cancer phytochemicals from* P. africana* can be divided into three major categories based on their targets and pharmacological effects (Table 1): (i) phytochemicals that kill the tumor cells through apoptotic pathways, a common mode of action of chemotherapeutic agents against a wide variety of cancer cells [60], (ii) phytochemicals that alter the signaling pathways required for the maintenance of prostate cancer cells, and (iii) phytochemicals that exhibit strong antiandrogenic and antiangiogenic activities. The details of the antiprostate cancer effects of each phytochemical are shown in Table 1.

### 5.1. Ursolic Acid

Ursolic acid (Table 1(a)) is a pentacyclic triterpene compound isolated from many types of medicinal plants and widely present in human diet [23, 28]. Several studies have suggested that ursolic acid is one of the main antiprostate cancer phytochemicals found in both root and stem bark extracts of* P. africana* [2, 22, 24, 25]. This acid has been suggested to suppress inflammation, reduce oxidative stress, regulate cell cycle, inhibit cell proliferation, induce apoptosis, and interact with the tumor microenvironment through modulation of multiple signal transduction pathways [22, 26]. A study has shown that the inhibition of cell viability and the induction of apoptosis in PC-3 and LNCaP cells by ursolic acid were associated with downregulation of the B-Cell Lymphoma 2 (BCL-2) protein [23], a member of the protein cell family that controls apoptosis to prevent prostate cancer progression [27, 28]. This crucially proves the ability of ursolic acid to treat human prostate cancer, a hormone-refractory and androgen-sensitive cancer [23], and to also inhibit the growth of prostate cancer cells [20]. Therefore, downregulation of BCL-2 by ursolic acid results in the apoptosis in human prostate cancer cells making the acid a suitable antiprostate cancer agent.

### 5.2. Oleanolic Acid

Oleanolic acid (Table 1(b)) is a naturally occurring pentacyclic triterpenoid found in both root and stem bark extracts of* P. africana* [2, 22, 24, 25]. This acid inhibits the survival and proliferation of prostate cancer cells through the induction of apoptosis and interacts with the tumor microenvironment through modulation of multiple signal transduction pathways [22, 26]. These observations were further confirmed by a study by Li et al. [29] who confirmed that oleanolic acid inhibited the cell viability and proliferation and promoted the cell apoptosis and* G*_0_/*G*_1_-phase cell cycle arrest in prostate cancer PC-3, DU145, and LNCaP cells, in a dose-dependent manner. The same study revealed that oleanolic acid exerted anticancer effects in vitro on PC-3 and DU145 cells by repressing the phosphoinositide 3-kinase (PI3K)/protein kinase B (Akt) pathway [29]. In another study, oleanolic acid exerted antitumor activity by interfering with a metabolic pathway in cancer cells through activation of the enzyme 5′-AMP-activated protein kinase (AMPK) [30]. These studies clearly demonstrated the anticancer properties of oleanolic acid for prostate cancer cells, both in vitro and in vivo, and provided the evidence for its use in further preclinical and clinical studies in prostate cancer patients.

### 5.3. Atraric Acid (AA)

Atraric acid is a naturally occurring phenolic compound and ester (Table 1(d)) found in the bark extract of* P. africana* [9, 32]. In vitro studies have shown that it has a very strong antiandrogenic activity [9, 32], which decreased the cell proliferation of PC-3-AR. However, the growth of PC-3 and non-PCa cells lacking the AR expression was not affected by AA, suggesting an AR-dependent growth inhibitory mechanism imposed by AA [32]. In another study which included a reporter gene assay, 10 *μ*M AA repressed the androgen AR-mediated transactivation by about 90% [32]. AA exhibited a 50% inhibition level at a concentration of 1 *μ*M but failed to repress the AR-mediated transactivation at 0.1 *μ*M. These findings show that AA represses androgen AR-mediated transactivation at higher concentrations. AA isolated from* P. africana* was found to exhibit similar inhibition of AR function to that of commercially available AA [6] which further signifies the importance of this phytochemical. AA molecules also inhibited AR translocation to the cell nucleus by binding to the receptor which resulted in downregulation of the androgen level [9]. AA also inhibited the cellular invasion of prostate cancer cells into the extracellular matrix, indicating that it may have a protective role against tumor invasion [33]. Furthermore, a study by Hessenkemper et al. [61] strongly showed that AA led to senescence-associated beta-galactosidase activity, an indication of cellular senescence that resulted into proliferation arrest in PC-3-AR. Therefore, the ability of AA to suppress AR and decrease PC-3 proliferation can be potentially employed in the prostate cancer chemoprevention and chemotherapy.

### 5.4. Ferulic Acid

Ferulic acid is an abundant phenolic phytochemical (Table 1(e)) found in plant cell wall components of many plants, including* P. africana* [20, 21]. Studies have shown that ferulic acid isolated from the bark extract of* P. africana* inhibited angiogenic pathways [20, 34] and consequently preventing the growth of new blood vessels from preexisting vessels, as well as the growth and spread of prostate cancer [35]. These results were somewhat supported by the data obtained by Mukherji et al. [37], who observed that the inhibition of angiogenic pathways was proven to be an effective strategy for the treatment of several common solid tumors although its definitive role in the management of prostate cancer is yet to be elucidated. In another study conducted by Eroğlu et al. [36], the results revealed that ferulic acid inhibited the cell proliferation and decreased the gene expression of BCL-2 an inhibitor of apoptosis protein 3 (IAP3) in LNCaP cells, resulting in the induction of apoptosis in PC-3 and LNCaP cells. In addition, the authors observed that ferulic acid suppressed the invasion of PC-3 and LNCaP cells [36]. The inhibition of angiogenic pathways and induction of apoptosis by ferulic acid makes it an important therapeutic agent for prostate cancer chemoprevention.

### 5.5. N-Butylbenzene-Sulfonamide (NBBS)

NBBS (Table 1(f)) is one of the compounds naturally found in the bark extract of* P. africana* [9, 32, 38]. It is a sulphur-containing compound that is widely used as a plasticizer in polyacetals and polyamides and it shows high antiandrogenic activity [9]. Although the NBBS phytochemical was found to decrease the cell proliferation of wild-type PC-3-AR cells, the growth of PC-3 and non-PCa cells lacking the AR expression was not affected, suggesting an AR-dependent growth inhibitory mechanism imposed by NBBS [32]. A reporter gene assay that compared AA and NBBS showed that NBBS was less efficacious as an inhibitor of androgen activated AR-mediated transactivation, exhibiting 90% repression only at a higher concentration (100 *μ*M) [9]. NBBS also inhibited both endogenous prostate-specific antigen (PSA) expression and growth of human prostate cancer cells [32]. NBBS molecules also inhibited AR translocation to the cell nucleus by binding to it, thereby downregulating the androgen level [9]. Although the limited available literature reports neurotoxicity of NBBS in rabbits [39], experiments with Sprague Dawley male rats showed no observable effects at a dose of 300 mg/kg/day administered for 27 days [38]. However, deaths of rats were observed at a higher dose of 400 mg/kg/day after 5 days of dosing [38]. These results were confirmed in a review study by Roell and Baniahmad [32] in which the authors revealed that NBBS only had a slight effect on rats at very high doses and a short duration of treatment and that* P. africana* extracts were well tolerated by humans. Therefore, the effects of NBBS on AR and subsequent decrease in PC-3 proliferation can be explored to maximize its potential for the chemoprevention of prostate cancer.

### 5.6. Beta-Sitosterol

Beta-sitosterol is a plant-derived sterol, also known as a phytosterol (Table 1(g)). The major effects of* P. africana* on prostate cancer have been reported to be due to the presence of this chemical which is present in high concentrations in the plant [24, 40]. Studies have shown that *β*-sitosterol affects the membrane structure and function of tumor as well as the host tissue signal transduction pathways that regulate tumor growth, leading to an apoptotic condition [40]. Furthermore, this phytosterol exhibited anti-inflammatory effects which suppressed the production of prostaglandins thereby preventing the swelling of the prostate [20]. It also inhibited the human prostate tumor cell invasiveness and reduced the release of matrix metalloproteinases [41]. Studies have also shown that *β*-sitosterol induced the apoptosis of LNCaP human prostate cancer cells [42, 43]. These antiprostate cancer activities exhibited by *β*-sitosterol can be further developed for the chemoprevention and chemotherapy of prostate cancer.

### 5.7. Lauric Acid

Lauric acid is a saturated medium-chain fatty acid with a 12-carbon backbone (Table 1(h)). It is found naturally in various plant and animal fats and oils and is one of the phytochemical components in the stem bark of* P. africana* [20]. It has been found that this acid inhibited the 5-*α*-reductase enzyme, thus preventing the formation of dihydrotestosterone, the modulator of prostate growth. [20]. Considering that dihydrotestosterone is implicated in the pathogenesis of prostate cancer [45], inhibition of the 5-*α*-reductase enzyme by lauric acid, resulting in the blockage of testosterone conversion to dihydrotestosterone, plays an integral role in the prevention and treatment of testosterone prostate cancer [44, 45]. The potential of lauric acid for chemoprevention of prostate cancer has been further supported by a study that showed its ability to inhibit the proliferation of LNCaP cells [46].

## 6. Use of* P. africana* in African Traditional Medicine to Treat Prostate Cancer

Despite the fact that the early detection and commencement of cancer treatment usually increase the chances of survival in developed world, the chances of survival in developing countries are usually much lower since the access to modern cancer diagnostic facilities and effective treatment methods are limited for most people especially for those living in rural areas. In Africa in particular, the herbalists rely on the use of potent herbal medicines to tackle the disease burden.* P. africana* is one of the plants frequently used in many African communities for the treatment of prostate cancer. Thus, according to a study by Ochwang'i et al. [1], the pounded stem bark of* P. africana* is usually mixed with water and drunk as a remedy for Prostate cancer in Kakamega county in Kenya.

## 7. Conclusion

Prostate cancer is still one of the leading causes of death in men worldwide. Therefore, all possible avenues have to be explored in an attempt to tackle the disease. Easily accessible and affordable options, other than the conventional methods of surgery, radiation therapy, cryosurgery, and hormone therapies especially for the poor need to be explored. This review proves that the use of naturally occurring phytochemicals from* P. africana* can be considered for both chemoprevention and chemotherapy of prostate cancer. This plant has a rich history of use in the treatment and management of prostate cancer in ATM. Scientific studies have proven that phytochemicals from* P. africana* have the ability to affect numerous targets associated with the degradation of the prostate cancer cells. Therefore, these pharmacological effects of* P. africana* phytochemicals can be exploited and utilized for the chemoprevention and chemotherapy of prostate cancer. However, more preclinical and clinical studies need to be done to validate these phytochemicals for possible use in the antiprostate cancer drug development. Unfortunately, despite its significant potential for the treatment of prostate cancer and other diseases, improper methods of stem bark harvesting and illegal logging of* P. africana* have made the plant an endangered species. As a result, there is need for advanced* P. africana* propagation techniques to urgently increase its population and distribution range beyond the current level so as to meet the ever increasing demand for this plant and its constituents.

## Figures and Tables

**Table 1:** In vitro and in vivo effects of *P. africana* phytochemicals on prostate cancer cells.

S/No	Class of compound	Phytochemical compounds	Compound structure	Cellular target	Cellular effects	Reference

(a)	Pentacyclic triterpenoid saponins	Ursolic acid	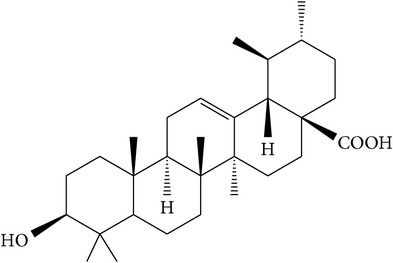	PC-3 LNCaP BCL-2	Inhibited growth of prostate cancer cells; downregulated Bcl-2	[2, 20, 22–28]

(b)	Pentacyclic triterpenoid saponins	Oleanolic acid	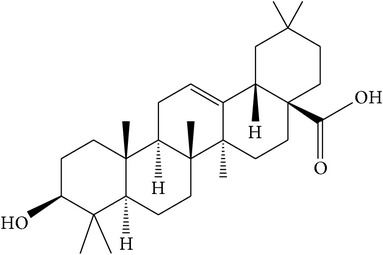	PC-3 DU145 LNCaP PI3K/Akt AMPK	Inhibited cell viability, proliferation, promoted cell apoptosis, and arrested *G*0/*G*1 phase cell cycle in prostate cancer cells	[2, 22–27, 29, 30]

(c)	Triterpene	*β*-amyrin	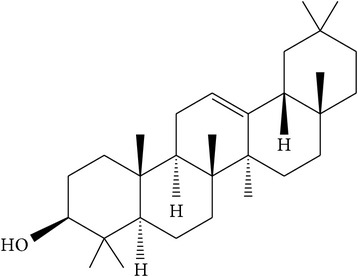	PC-3	Exhibited cytotoxicity and apoptosis on prostate cancer cells	[31]

(d)	Phenol	Atraric acid	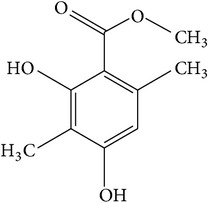	PC-3-AR AR	Showed anti-androgenic activity, inhibited AR translocation to the cell nucleus, decreased cell proliferation of PC-3-AR, and inhibited cellular invasion by prostate cancer cells into the extracellular matrix	[6, 9, 32, 33]

(e)	Phenol	Ferulic acid	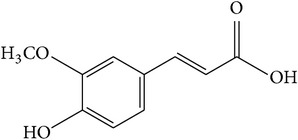	BCL-2 LNCaP PC-3	Inhibited the angiogenic pathways, inhibited cell proliferation, and promoted apoptosis of PC-3 and LNCaP by downregulating Bcl2 expression	[20, 21, 34–37]

(f)	Phenol	N-butylbenzene-sulfonamide	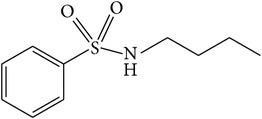	PC-3-AR AR	Showed antiandrogenic activity, decreased the cell proliferation of PC-3-AR, and inhibited AR translocation to the cell nucleus	[9, 32, 38, 39]

(g)	Sterols	*β*-sitosterol	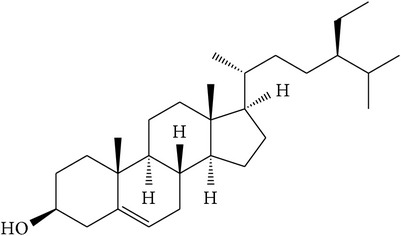	LNCaP	Exhibited cytotoxicity and apoptosis and suppressed the production of prostaglandins	[2, 15, 20, 24, 31, 40–43]

(h)	Fatty acid	Lauric acid	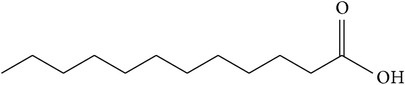	LNCaP	Inhibited 5-*α*-reductase enzyme thus preventing formation of dihydrotestosterone, the modulator of prostate growth	[44–46]

Molecular structure source: https://en.wikipedia.org/.

PC-3: human prostate cancer cell lines. LNCaP: lymph node carcinoma of prostate cell line. BCL-2: B-cell lymphoma 2 protein, which regulates cell death (apoptosis). DU145: a cell line of prostatic cancer derived from brain metastasis. PI3K/Akt: phosphoinositide 3-kinase/protein kinase B, which regulates multiple biological processes including cell survival, proliferation, growth, and glycogen metabolism. AMPK: 5′ adenosine monophosphate-activated protein kinase, an enzyme that plays a role in cellular energy homeostasis. AR: androgen receptor which translocates androgen hormones to the nucleus. PC-3-AR: Human prostate cancer cell lines that expressed the androgen receptor.

## Additional Points

*Limitations*. This review has some limitations. First, during our review process we did not include unpublished information and hence our findings could be affected by a publication bias. Second, we might have missed studies or vital information published on sites other than those we focused on. Nevertheless, this review is very significant in that it clearly reveals the potential of* P. africana* for the chemoprevention and chemotherapy of prostate cancer.
